# Capacity challenges in water quality monitoring: understanding the role of human development

**DOI:** 10.1007/s10661-020-8224-3

**Published:** 2020-04-19

**Authors:** Sabrina Kirschke, Tamara Avellán, Ilona Bärlund, Janos J. Bogardi, Laurence Carvalho, Deborah Chapman, Chris W. S. Dickens, Kenneth Irvine, SungBong Lee, Thomas Mehner, Stuart Warner

**Affiliations:** 1 United Nations University - Institute for Integrated Management of Material Fluxes and of Resources (UNU-FLORES), Dresden, Germany; 2 Helmholtz-Centre for Environmental Research, Magdeburg, Germany; 3 University of Bonn, Bonn, Germany; 4 UK Centre for Ecology & Hydrology, Penicuik, UK; 5 University College Cork, Cork, Ireland; 6 International Water Management Institute, Colombo, Sri Lanka; 7 IHE Delft Institute for Water Education, Delft, Netherlands; 8 Wageningen University & Research, Wageningen, Netherlands; 9 Leibniz-Institute of Freshwater Ecology and Inland Fisheries, Berlin, Germany

**Keywords:** Capacity development, Global survey, Human development index, SDG 6, Water quality parameters

## Abstract

Monitoring the qualitative status of freshwaters is an important goal of the international community, as stated in the Sustainable Development Goal (SDGs) indicator 6.3.2 on good ambient water quality. Monitoring data are, however, lacking in many countries, allegedly because of capacity challenges of less-developed countries. So far, however, the relationship between human development and capacity challenges for water quality monitoring have not been analysed systematically. This hinders the implementation of fine-tuned capacity development programmes for water quality monitoring. Against this background, this study takes a global perspective in analysing the link between human development and the capacity challenges countries face in their national water quality monitoring programmes. The analysis is based on the latest data on the human development index and an international online survey amongst experts from science and practice. Results provide evidence of a negative relationship between human development and the capacity challenges to meet SDG 6.3.2 monitoring requirements. This negative relationship increases along the course of the monitoring process, from defining the enabling environment, choosing parameters for the collection of field data, to the analytics and analysis of five commonly used parameters (DO, EC, pH, TP and TN). Our assessment can be used to help practitioners improve technical capacity development activities and to identify and target investment in capacity development for monitoring.

**Electronic supplementary material:**

The online version of this article (10.1007/s10661-020-8224-3) contains supplementary material, which is available to authorized users.

## Introduction

Water quality has deteriorated from human activities such as urban wastewater disposal, agriculture and industrial production. Today, poor water quality is widespread and has a major negative effect on many aspects of human society, economy, ecology and the environment (WWAP 2012; UN Water 2018a, b). This has led the international community to set goals to restore the good qualitative status of freshwater resources. At a global level, such goals have generally been laid down in principled agreements that are voluntary without any sanction, the prime example being the Sustainable Development Goal (SDG) 6 on water and the respective target 6.3 on improving water quality by 2030 (E/CN.3/2017/2). At a regional level, regulations, which are backed by legal sanctions, are in place in some regions, for example the EU through the European Water Framework Directive (CEC 2000).

Implementing such goals require sophisticated water quality monitoring programmes, based on water quality parameters that are both relevant and measurable. Through the accepted UN SDG indicator 6.3.2, committed parties have agreed to an assessment of their freshwaters using the metric ‘proportion of bodies of water with good ambient water quality’. To measure this, a set of parameter groups and specific parameters are used as representative of the state of a water body, which can be assessed relatively easily using established methods. The parameter groups used are oxygen (dissolved oxygen, biological oxygen demand, chemical oxygen demand), salinity (electrical conductivity, salinity, total dissolved solids), nitrogen (total oxidized nitrogen, total nitrogen, nitrite, ammonia, nitrogen, nitrate), phosphorus (orthophosphate, total phosphorous) and acidification (pH) (UN Water 2018a, b).

Experience has shown that monitoring is impeded because data on such parameters are missing at the relevant temporal and spatial scales (AbuZeid et al. 2014; EEA 2012; Evans et al. 2012; UNEP 2010; UNEP 2016; UN Water 2018a, b). The first reporting period of SDG indicator 6.3.2 (the UN Water organized data drive in 2017, including data from that year or the preceding 3 years 2014–2017), for instance, has shown that data are either dispersed or lacking in certain parts of the world, even for apparently ‘simple to measure’ parameters such as pH (UN Water 2018a, b). Data deficiencies exist for the related fields of water, sanitation and hygiene, where we find insufficient quantity and quality of reported data (UNICEF/WHO 2019; UN Water 2019; WHO/UNICEF 2017). So, the challenge that strikes both scientific monitoring specialists and UN entities that support such monitoring activities is how this water quality data gap can be closed at a global level (UNEP 2016; UN Water 2018a, b).

Research on water quality monitoring has identified a set of factors influencing the success of monitoring programmes. Prominent amongst these is the capacity of monitoring agencies, including aspects related to human capacity (e.g. staff retention, knowledge, motivation and leadership), funding of monitoring activities and the availability of technical equipment (e.g. Beck et al. 2010; Behmel et al. 2016; Delaire et al. 2017; Ferrero et al. 2019; Irvine et al. 2016; Peletz et al. 2018; Rahman et al. 2011; Steynberg 2002; UN Water 2019). Most of these water quality monitoring studies focus, however, on specific uses such as drinking water (e.g. Ferrero et al. 2019; Steynberg 2002) or specific regions such as Africa (e.g. Delaire et al. 2017; Peletz et al. 2018), Europe (Beck et al. 2010; Poikane et al. 2015), developing countries in general (Rahman et al. 2011) or the tropics (Irvine et al. 2016).

However, research on water quality monitoring does not consider systematically the context of *human development* in which such monitoring activities take place. By considering both financial and educational aspects, for instance, the concept of human development and related indices hint to the capacity of states to provide public services. By consequence, human development is also likely to influence the lack or availability of capacity and thus also the effectiveness of monitoring processes. Human development considers, for instance, economic growth, which is likely to influence the financial resources available for monitoring freshwater resources. Human development also indicates (implicitly) the knowledge base of societies that may influence the availability of skilled personnel for monitoring. Thus, while it has been largely neglected in monitoring discussions so far, human development is an important contributory factor to monitoring challenges even for ‘easy-to-measure’ parameters and deserves some systematic analysis.

Against this background, the goal of this study was to add to the planning of successful water quality monitoring, by analysing systematically the impact of human development on the capacity for water quality monitoring within the monitoring cycle—from defining an enabling environment (existence of obligatory rules, monitoring strategies, responsible authorities and institutional capacity), to the selection of relevant water quality parameters and then to actual measurement of five representative water quality parameters: dissolved oxygen (DO), electrical conductivity (EC), pH, total phosphorus (TP) and total nitrogen (TN). To understand capacity challenges along the monitoring cycle, we conducted a survey amongst international water quality experts from science and practice in the final phase of the first SDG 6.3.2 data drive in 2017. We then further analysed the role of human development in such water monitoring capacity challenges by using the corresponding data of the human development index (HDI). The HDI admittedly characterizes human development, aggregating not only information on the standard of living (based on gross national income per capita), but also on health and knowledge (including here expected and mean years of schooling and life expectancy). This turns the HDI into a particularly comprehensive and relevant influencing factor for capacity challenges in water quality monitoring (UNDP 2016).

The next section clarifies the concepts of ‘human development’ and ‘capacity challenges in water quality monitoring’ and develops the relationship between the two. We then describe the methods for gathering and analysing the data, focussing on the methodology applied in the international survey amongst water quality experts. The following section then presents the results of the analysis, starting with descriptive data related to the HDI and capacity challenges, and then dealing with the correlation between the HDI and capacity challenges along the application process. The final section discusses and concludes on the results, putting emphasis on targeted strategies to overcome identified capacity challenges in water quality monitoring.

## Human development and capacity challenges in water quality monitoring: concepts and relationships

### Concepts

In terms of *human development*, there exist a high number of indicators, which generally capture two factors of human development: welfare and participation (Neuenfeldt et al. 2012). In this analysis, however, we apply the human development index (HDI) because it aggregates different dimensions of human development, which refer to health, knowledge and the standard of living. These dimensions are measured based on the indicators of life expectancy, expected and mean years of schooling and gross national income per capita (UNDP 2016). As such, the HDI is likely to have a particularly strong relationship with the monitoring of water quality. This relates to the crucial contribution of both financial resources and the formal level of education because financial resources are vital to fund human resources as well as relevant equipment for water quality monitoring, and the formal level of education is likely to influence the existence of skilled human resources for monitoring. Moreover, the HDI is quite commonly considered in the field of water management, e.g. to measure the impact of human development on Integrated Water Resources Management (IWRM) (see UNEP 2012).

**Fig. 1:**
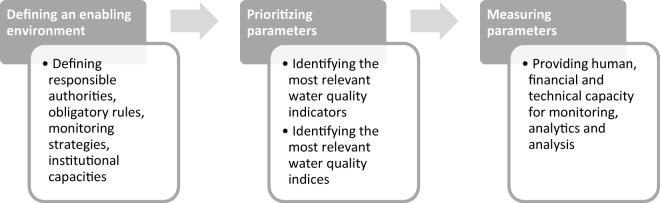
Potential capacity challenges of water managers in Water Quality Monitoring along the monitoring process

*Capacity challenges in water quality monitoring* can refer to different types of difficulty in water quality monitoring, such as different geographical contexts or unclear (i.e. controversially discussed) beneficial uses in societies. Given the focus of this study, we apply here a more specific understanding of capacity challenges, referring to a *lack of capacity (or ability)* of water managers (organisations and individuals) in water quality monitoring along the monitoring cycle—from defining the enabling environment (existence of responsible authorities, obligatory rules, monitoring strategies and institutional capacity to measure water quality), continuing with choosing the right indicators and indices^1^ at an organisational level (identifying the most relevant water quality parameters and sets of parameters within monitoring strategies), right through to the actual measurement of parameters (including human, financial and technical capacity for monitoring, analytics and analysis).^2^ Capacity challenges can be general (general challenges in defining an enabling environment or prioritizing parameters) or specific (financial, human or technical issues faced in measuring parameters) (Fig. 1). They are conceptualized here as ordinal variables (different degree of challenges, e.g. more or less challenges in identifying the most relevant water quality indicator) (see in more detail the “Methods” section). Conceptualizing capacity deficits in these different phases of the measurement cycle is helpful since challenges may vary between these phases (see below). The different phases are also congruent with the multi-level approach to water-related capacity development (Leidel et al. 2012; Wehn de Montalvo and Alaerts 2013; Ibisch et al. 2016), which may help to systematically conceptualize measures for capacity development along these phases.

### Relationship between HDI and capacity challenges in water quality monitoring

Relationships between the HDI and capacity challenges in water quality monitoring are discussed based on a rational approach to decision-making. Such an approach considers the costs and benefits of individuals and organisations in decision-making with regard to water quality monitoring. Both benefits and costs can be material (i.e. increase or decrease of financial resources) and immaterial (e.g. loss or gain of time, reputation). The perceived benefits of a decision can both exceed and fall below the costs associated with the decision. We discuss the cost-benefit ratio with regard to the general relationship between the HDI and capacity challenges in water quality monitoring and with regard to the specific relationship between the HDI and capacity challenges in water quality monitoring in relation to the monitoring process. The general idea here is that decisions to define an enabling environment, identifying indicators and indices as well as on measuring specific parameters can be associated with both costs and benefits which again influence if a decision is taken.

We first consider the general relationship between the HDI and capacity challenges in water quality monitoring. We assume a negative relationship between the two variables, with benefits exceeding the costs in high HDI countries in contrast to costs exceeding the benefits in low HDI countries. Such a negative relationship is justified because high HDI countries, with higher gross national income (GNI) per capita, may have lower relative costs for water quality monitoring in comparison with low HDI countries (UNDP 2016). At the same time, there are arguments to suggest that perceived benefits of water quality monitoring may be higher in high HDI countries than in low HDI countries: First, we may expect that in less-developed countries, there is less knowledge in the general public of the benefits of good water quality given lower education levels. Second, even if we do not expect strong differences in environmental concern in low or high HDI countries as recent research has suggested (Dunlap and York 2008; Marquart-Pyatt 2012), the current need to comply with comprehensive regulatory frameworks in the field of water quality management may increase high HDI countries’ perception of benefits of water quality monitoring (e.g. CEC 2000; Carvalho et al. 2019).

Besides, high HDI countries may have better skilled human resources for water quality monitoring, given generally higher education rates (UNDP 2016). Moreover, environmental performance in terms of environmental health and ecosystem vitality seems to increase in developed countries (Samimi et al. 2011). In water management, for instance, the concept of Integrated Water Resources Management seems to be better implemented in developed countries than in less-developed countries even though developing countries have put particular emphasis on promoting the concept (Leidel et al. 2012: 1425; UNEP 2012).

- *Assumption 1* The higher the HDI, the less capacity challenges exist in water quality monitoring.

As a second step, we consider the specific relationship between the HDI and capacity challenges in water quality monitoring in relation to the monitoring process. We assume a negative relationship between the HDI and capacity challenges along the steps of monitoring, based on the perceived cost-benefit ratio of water quality monitoring. A ‘perceived cost-benefit ratio’ means that water managers may, based on their subjective view, attribute specific costs and benefits to water quality monitoring activities. Water managers may, more particularly, assume increasing costs and decreasing benefits of water quality monitoring in the course of the monitoring process. In terms of costs, the following may be stated:

- At the very beginning of the process, establishing definitions of a so-called enabling environment (e.g. defining rules for measuring water quality, identifying a responsible authority for measuring, defining management plans and strategies) may be comparatively less challenging than the actual enforcement of rules, plans and strategies for measuring water quality. This assumption is based on the empirical abundance of water quality regulations and guidance on the one hand, and a lack of monitoring data provided by such environments on the other hand (e.g. Steynberg 2002; Rahman et al. 2011; UN Water 2018a, b).
- By comparison, the second step in the application process—enforcement of the monitoring rules—seems more challenging. Enforcing rules for measuring water quality initially requires identification of the right set of parameters. This seems challenging, particularly for poorly equipped enabling environments, mainly due to the large number of water quality indices that are available. A quick review of nine international and national guidelines on water quality including the WHO guidelines on drinking water quality (WHO 2011) shows, for instance, that there is a large set of almost 500 parameters to measure the qualitative status of waters (see Annex 1). In addition, research has produced great diversity in terms of water quality indices (Abbasi and Abbasi 2012; Bharti and Katyal 2011). Whereas this abundance is a great achievement, it also confronts water managers with an important selection problem since there are increasingly limited resources to measure parameters (Horowitz 2013) and not all parameters are appropriate for a given context (UN Water 2015; Irvine et al. 2016).
- Finally, the third step in the application process—the actual enforcement of rules by implementing the monitoring of parameters including their measuring and respective analytics and analysis (see above) is particularly cost-intensive given the need for both skilled personal and laboratory and field infrastructure. This may, in particular, challenge poorly equipped environments in their water quality monitoring, so that the written regulations which simulate a functioning enabling environment are not effective in ensuring monitoring takes place (e.g. Peletz et al. 2018).

At the same time, the benefits may also vary along the process, given the varied visibility to the public of the various actions. Taking care of legal rules can be easily interpreted by the public as an effective measure to address poor water quality, while choosing and applying the appropriate set of parameters is more technical and not so straightforward to the public. Choosing and applying the right set of parameters may thus not be prioritized by public authorities if costs for water monitoring are relatively high.

Thus, it might be expected that both high and low HDI countries may deal with the first step, whereas the implementation of the choice of water quality parameters and the actual measurement, may be considerably more challenging for low HDI countries.

- *Assumption 2* The relationship between the HDI and capacity challenges in water quality monitoring varies along the monitoring process, with a rather low impact of the HDI at the beginning of the process (defining an enabling environment) and a rather strong impact of the HDI at the end of the process (measuring parameters).

## Methods

### Data gathering

To test the assumptions, between May and September 2017, we conducted an experts’ survey on capacity challenges in water quality monitoring. The survey was accessible in the six official UN languages (Arabic, Chinese, English, French, Russian and Spanish), following a link provided on the UNU-FLORES homepage (UNU-FLORES 2017). Links to the survey were spread amongst different networks of water quality experts (see Annex 2), amongst them networks of scientists (mainly natural scientists) and practitioners (mainly water utilities and public authorities), at an international level (involving countries of different regions, covering countries from low to high human development), as well as at a regional (e.g. European countries, Arab countries) and national level (Germany and Australia).

The survey was structured with four main categories: Section 1 on general information served to generate background information of the respondents and the specific case the respondents referred their answers to. This also included information on the specific country the respective case referred to. Related questions were closed and answer categories were on a nominal scale. Section 2 referred to the general use of parameters, and aimed at clarifying purposes, challenges, standards and the application of different types of parameters. Related questions were mainly closed, and mostly offered answers on a 1–4 scale (1 = not challenging/relevant–4 = very challenging/relevant), with an additional opportunity for a ‘do not know’ answer. In the subsequent Section 3, we aimed at specifying capacity challenges related to the application of the five commonly used parameters DO, EC, pH, TP and TN (Srebotnjaka et al. 2012; UN Water 2018a, b). Related questions identified challenges on a 1–4 scale (1 = not challenging–4 = very challenging), with an additional opportunity for a ‘do not know’ answer. Finally, Section 4 was on management and governance challenges related to the monitoring of water quality parameters, which refer here to our idea of an enabling environment for water quality monitoring. Related questions were mainly closed, with ‘yes’, ‘no’ and ‘do not know’ answers hinting to the existence of respective challenges. Annex 3 provides a detailed list of all the questions and response categories.

A total of 114 respondents completed the online questionnaire. Out of these, 104 were valid and analysed since the remaining questionnaires were either blank (4 times) or did not contain any information on the country the answers referred to (6 times). Within the 104 valid questionnaires, the respondents answered most of the questions by either giving a qualitative answer or a ‘do not know’ answer, with an average percentage of answers of 91.3% and a standard deviation of 9.0%. Thus, only a small number of questions were left out completely blank by the 104 respondents. For this analysis, we generally provide percentages and averages based on the total number of qualitative answers per question. This absolute number of answers is indicated in Annex 4.

Respondents of the questionnaire represent mainly government officials (45.2%) and academics (30.8%), followed by a limited number of actors from the private sector (9.6%), civil society (5.8%), development agencies (2.9%) and international organisations, public utilities and other sectors (together 5.8%). Experience related to water quality parameters was high, with the majority indicating more than 7 years of experience (68.6%), about a quarter indicating 4 to 6 years of experience (21.6%) and only a small number indicating less than 3 years of experience (9.8%). Respondents represented 53 different nationalities. There were slightly more male respondents (56.3%) than female (43.7%). Most respondents provided a personal answer (88.5%) and a minority provided an answer for a group such as for the organisation they work with (11.5%).

The answers, from 57 different countries (see in more detail Section 4), are greatly congruent with the nationality of the respondents, meaning that the countries the respondents referred their answers to were mostly congruent with the actual nationality of the respondents. Answers also mostly referred to a specific water body (35.6%) followed by the regional level (27.9%) and (sub)-basin level (16.4%), with 20.2% of answers addressing another scale which they further specified (mostly national level, but also specific areas such as the river-sea continuum). In terms of the types of water, most of the answers referred to rivers (44.3%) followed by lakes (27.3%) and groundwater (18.6%). Only a minority included estuaries (9.8%). Moreover, beneficial uses of water were considered, with the provision of drinking water being of greatest relevance for addressing water quality concerns (Table 1).

**Table 1:** Beneficial uses of water. Depicted are mean values for six types of beneficial uses as described in the US Clean Water Act (Federal Water Pollution Control Act 2008). Scale from 1 (not important) to 4 (very important)

No	Beneficial use	Mean
1	Public water supplies (referring to providing drinking water)	3.4
2	Agricultural use (referring to the cultivation of crops for food and energy supply)	3.1
3	Propagation of fish, shellfish and wildlife (referring to fishing purposes and biodiversity)	2.9
4	Recreation in and on the water (referring to tourism)	2.7
5	Industrial use (referring to the production of various goods)	2.5
6	Navigation (referring to the transport of goods and people)	1.8

### Data analysis

The statistical analysis integrates all valid answers per question excluding any ‘do not know’ answers. In terms of the enabling environment, these are all ‘yes’ and ‘no’ answers for the four sub-variables. In terms of the phase of prioritization and the three sub-phases of the measurement process, these are all 1–4 answers, representing different degrees of challenges (from 1 = not challenging up to 4 = very challenging). To analyse the data, answers related to the same country were averaged, resulting in a total set of 57 country cases for the descriptive and subsequent correlation analysis. Those country cases were assigned specific HDI levels based on official data related to the reporting period (UNDP 2016). To analyse the relationship between the HDI and measurement challenges, the Spearman rank correlation *r*_Sp_ was used, given the interval scale of the independent variable and the ordinal scale of the dependent variables, as well as the non-normal distribution of the data set.

## Results

### Descriptive data

The descriptive analysis describes both the distribution of the HDI and capacity challenges. Data is available in Annex 4.

### Independent variable: Human Development Index

**Fig. 2:**
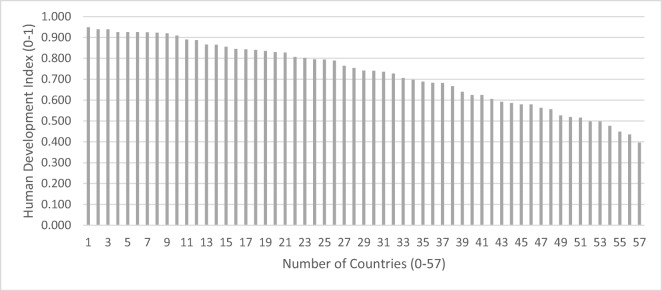
Human Development Index related to 57 cases (i.e. condensed country-specific points of reference as described in the 104 valid questionnaires)

The Human Development Index (HDI) of the cases (i.e. country-specific points of reference as described in the 104 valid questionnaires) ranged from 0.40 to 0.95. Considering the 57 countries, the HDI was 0.73 on average, with a standard deviation of 0.16 (Fig. 2). This result only differs slightly from the analysis across the 104 answers, which shows an average HDI of 0.77 and a standard deviation of 0.17.

**Fig. 3:**
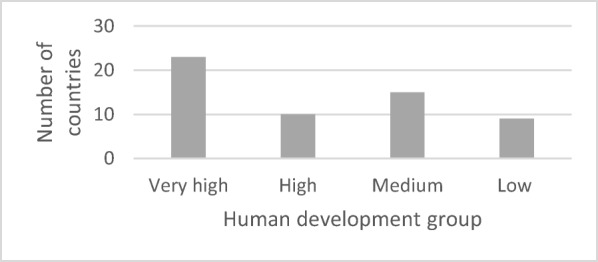
Number of country cases per human development group, based on 57 country cases

The HDI rank of the countries ranges from rank 1 (Norway, with very high human development, HDI = 0.949) to 186 (Chad, with low human development, HDI = 0.396). The countries thus represent almost the entire range of possible HDI ranks from 1 (very high human development, HDI = 0.949) to 188 (low human development, HDI = 0.352) (UNDP 2016). Moreover, most of the countries in our data set of 57 countries have a very high human development (23 answers, HDI ≥ 0.8, e.g. Norway, Australia and Switzerland). A smaller number of countries have a high human development (10, HDI ≥ 0.7, e.g. Uruguay, Bulgaria and Malaysia), medium human development (15, HDI ≥ 0.55, e.g. Zambia, Cambodia and Myanmar) or a low human development (9, HDI < 0.55, e.g. Ethiopia, Congo and Chad) (Fig. 3).

### Dependent variable: capacity challenges in water quality monitoring

#### Enabling environment for measuring water quality

**Fig. 4:**
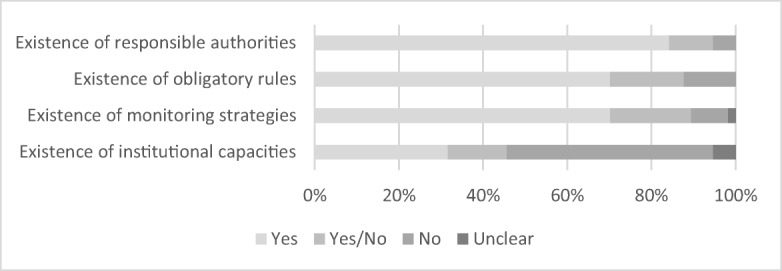
Enabling environment to measure water quality, based on 57 country cases

The enabling environment is determined by the existence of responsible authorities, obligatory rules, monitoring strategies and institutional capacity to measure water quality. Respondents affirmed here for most of the countries the existence of an enabling environment, clearly stating the existence of responsible authorities (84.2%), obligatory rules (70.2%) and monitoring strategies (70.2%). Only for a few countries, the indicators for an enabling environment were clearly negated. For the existence of institutional capacity, however, 49.1% of respondents stated that capacity did not exist, identifying a clear deficit whereas 31.6% assumed these did exist. For a small number of cases, respondents gave both ‘yes’ and ‘no’ answers, meaning that different respondents evaluated the indicators for an enabling environment differently. The country-specific information was generally adequate, with a few missing answers only (Fig. 4).

#### Prioritization of parameters

**Fig. 5:**
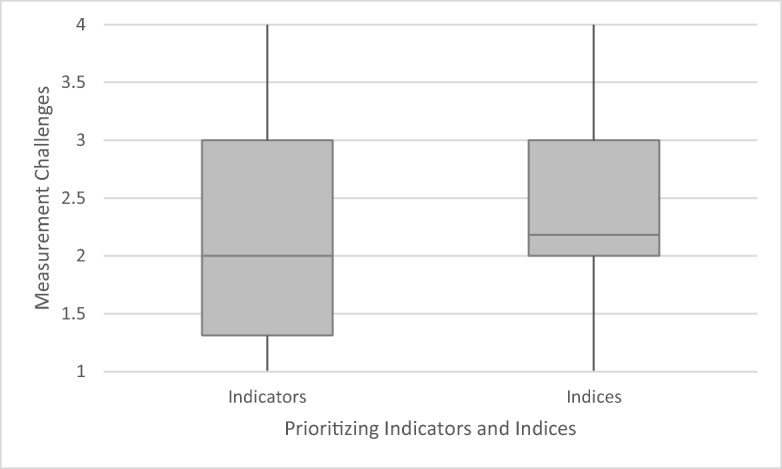
Difficulty to identify the most relevant water quality indicators and indices based on 57 country cases. Middle lines indicate the median, grey areas the quartiles and vertical lines minimum and maximum values

Prioritization is measured on a scale of difficulty (from 1 = not difficult to 4 = very difficult) to identify the most relevant water quality indicators and indices. Water quality indicators are here understood as water quality parameters, and indices refer to specific combination of parameters. Based on the 57 countries, difficulties to identify suitable indices and indicators are modest (median index = 2.2; median indicators = 2.0). Figure 5 builds on these results, demonstrating the median, lower and upper quartiles as well as the range of answer categories.

#### Actual measurement of parameters

Measurement challenges were analysed for five key parameters TN, EC, TP, DO and pH and measured along a 4-point scale (from 1 = not challenging to 4 = very challenging). Table 2 presents the median values for each parameter and category of measurement based on the 57 countries.

**Table 2:** Capacity challenges in applying the parameters DO, EC, pH, TP and TN based on 57 country cases^*^

Measurement challenges		DO	EC	pH	TP	TN
Monitoring	Technical equipment	2.0	2.0	1.8	2.0	2.0
	Human skills	2.0	2.0	2.0	2.4	2.3
	Financial means	2.8	2.1	2.0	3.0	3.0
Analytics	Technical equipment	2.2	2.0	1.8	3.0	3.0
	Human skills	2.0	2.0	2.0	2.0	2.3
	Financial means	3.0	2.3	2.0	3.0	3.0
Data handling/analysis	Technical equipment	2.0	2.0	2.0	2.0	2.1
	Human skills	2.0	2.0	2.0	2.0	2.0
	Financial means	3.0	2.5	2.2	2.8	3.0
	Transferring data	2.6	2.5	2.0	2.8	2.8

^*^Scale was from 1 (not challenging) to 4 (very challenging). Depicted are median values per category of measurement and parameter

**Fig. 6:**
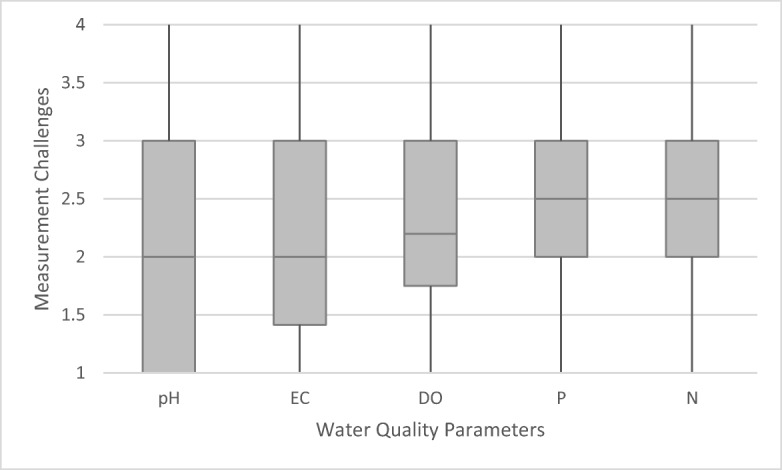
Measurement challenges of five water quality parameters averaged over the 10 measurement challenges as indicated in Table 2, based on 57 country cases. Middle lines indicate the median, grey areas the quartiles and vertical lines minimum and maximum values

**Fig. 7:**
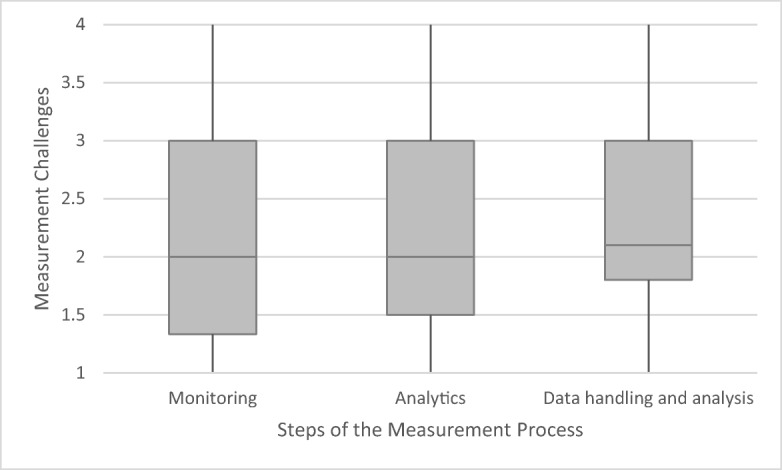
Measurement challenges along the steps of the measurement process as indicated in Table 2, based on 57 country cases. Middle lines indicate the median, grey areas the quartiles and vertical lines minimum and maximum values

**Fig. 8:**
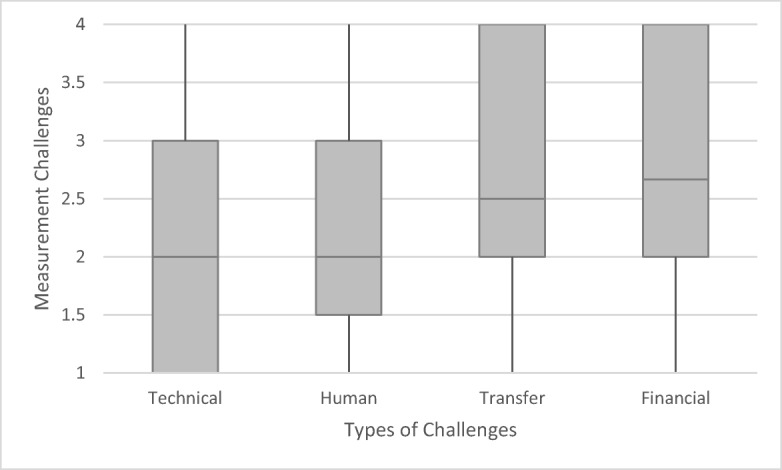
Measurement challenges along types (challenges regarding technical equipment, human skills, financial means, transferring data), as indicated in Table 2, based on 57 countries. Middle lines indicate the median, grey areas the quartiles and vertical lines minimum and maximum values

All in all, the degree of challenge identified by the respondents is moderate over all parameters and measurement categories. However, there are slight variations between the single parameters and categories of measurement (phases of measurement and types of measurement challenges), as a more in-depth comparison of medians, lower and upper quartiles, as well as minimum and maximum values shows. A direct comparison of the five parameters shows that they all tend to entail moderate degrees of measurement challenge, with slightly higher values for P and N (Fig. 6). A further comparison of the three different steps of measurement shows that measurement challenges are moderate in all three steps of the measurement process, with a particularly small increase along the process from monitoring and analytics to data handling and analysis (Fig. 7). Types of challenges (e.g., technical equipment, human skills and financial means) tend to be modest: here, challenges with regard to the provision of human skills and technical equipment were perceived as lower than providing financial means and transferring data (Fig. 8).

### Relationship between HDI and capacity challenges

#### Relationship between HDI and indicators for an enabling environment

Relations between the HDI and indicators for challenges with regard to the enabling environment are consistently negative. However, negative correlations between the HDI and challenges with regard to the existence of responsible authorities, obligatory rules and monitoring strategies are low and not significant. By contrast, lower HDI corresponds with low institutional capacities in a more pronounced way, which is also significant at a 0.01 error level (Table 3).

**Table 3:** Correlations between HDI and indicators for challenges with regard to the enabling environment. Depicted are correlations based on Spearman rank correlations (upper line) as well as error probabilities (lower line)

HDI value	Existence of responsible authorities	Existence of obligatory rules	Existence of monitoring strategies	Existence of institutional capacities
*r*_Sp_	− .175	− .171	− .217	− .449^**^
*p*	.192	.202	.108	.001

^**^The correlation is significant at the .01 level (two-tailed)

#### Relationship between HDI and prioritization of parameters

Relations between the HDI and challenges regarding prioritizing indices and indicators are again negative. However, these are only negative to a low degree and not significant (Table 4).

**Table 4:** Correlations between HDI and difficulties to identify the most relevant indicators and indices. Depicted are Spearman rank correlations (upper line) and error probabilities (lower line)

HDI value	Difficulty to identify the most relevant water quality indicator	Difficulty to identify the most relevant water quality index
*r*_*Sp*_	−.245	−.113
*p*	.068	.417

#### Relationship between HDI and the measurement of parameters

Relations between the HDI and measurement challenges are again negative, to different degrees, and mostly significant at a 0.01 error level (Table 5). This negative relationship is the strongest for *N* (mean = − 0.48), followed by *P* (mean = − 0.47), EC (mean = − 0.40), DO (mean = − 0.39) and pH (mean = − 0.37). Moreover, the relationship is slightly stronger for analytics (mean = − 0.44), followed by data handling and analysis (mean = − 0.42) and monitoring (mean = − 0.40). Finally, this relationship is the strongest for financial resources (mean = − 0.49), followed by technical equipment (mean = − 0.46), transferring data (mean = − 0.38) and human resources (mean = − 0.33).

**Table 5:** Correlations between HDI and measurement challenges. Depicted are Spearman rank correlations and the level of significance

HDI Value	Measurement challenges		DO	EC	pH	TP	TN
	Monitoring	Technical equipment	− .392**	− .335*	− .311*	− .467**	− .570**
		Human skills	− .326*	− .288*	− .188*	− .354**	− .389**
		Financial means	− .434**	− .419**	− .463**	− .501**	− .495**
	Analytics	Technical equipment	− .566**	− .460**	− .405**	− .578**	− .591**
		Human skills	− .404**	− .369**	− .187	− .355**	− .369**
		Financial means	− .394**	− .498**	− .468**	− .498**	− .442**
	Data handling/analysis	Technical equipment	− .388**	− .430**	− .384**	− .483**	− .511**
		Human skills	− .222	− .361**	− .387**	− .381**	− .395**
		Financial means	− .492**	− .591**	− .471**	− .580**	− .549**
		Transferring data	− .262	− .288*	− .412**	− .458**	− .460**

^*^The correlation is significant at the .05 level (two-tailed)

^**^The correlation is significant at the .01 level (two-tailed)

## Discussion and conclusion

Our analysis reveals notable negative correlations between human development and measurement challenges which increase over the course of the measurement process. This generally supports our hypotheses on the link between HDI and capacity for water quality monitoring, based on a rational approach to decision-making in water quality monitoring.

In hypothesis 1, we assumed a negative relationship between the HDI and capacity challenges in water quality monitoring. The consistently negative correlations between the HDI and measurement challenges, that are also mostly significant at the 0.01 error level, support this hypothesis. However, very weak and non-significant negative correlations with regard to the existence of responsible authorities, obligatory rules and monitoring strategies as well as with the difficulty to identify the most relevant water quality index and indicators extenuate this pattern. This suggests that human development may not necessarily affect the main foundations for monitoring water quality (by setting up a responsible authority to implement an obligatory rule and monitoring plan, based on existing indices and indicators), while it may rather influence the extent to which responsible actors struggle with the implementation of associated monitoring goals (owing to lack of institutional capacity to define monitoring strategies and actually monitor water quality) in the considered cases. Considering that the perceived costs and benefits may influence decisions, a reason may be that higher overall gross national income (GNI) per capita of high HDI countries (UNDP 2016) leads to lower relative costs and higher related benefits from water quality monitoring, compared with low HDI countries. This can also be influenced by the more recent need for high HDI countries to comply with regulatory frameworks exemplified by the EU water Framework Directive 2000/60/EC.

In hypothesis 2, we expected a rather low impact of the HDI at the beginning of the process (defining an enabling environment) and a rather strong impact of the HDI at the end of the process (measuring parameters). We find here that the strength of the negative correlation increases in the course of the measurement process, with more neutral to slight negative correlations in terms of defining the enabling environment and prioritizing relevant water quality parameters, and stronger negative correlations regarding measurement challenges. One exception is the enabling factor ‘existence of institutional capacity’ which is quite strongly negatively correlated with the HDI, significant at the 0.01 error level. Assuming again that the perceived costs and benefits may influence decisions, one potential reason could be that setting up responsible authorities and obligatory rules may be less cost-intensive and more prestigious than equipping such authorities with the means to also implement their mandate, so that particularly low-income countries would fall short in this capacity-related factor.

While there is some evidence substantiating our hypotheses, we also see limitations of our study which may bias our results. First, we acknowledge a limited conceptualization of ‘capacity challenges’. This relates, for instance, to our understanding of an ‘enabling environment’, which may also include qualitative aspects (e.g. the existence of precise non-contradictory rules), in addition to the sheer definition of water monitoring rules (i.e. existence of a rule as such). Second, we acknowledge a somewhat small data sample, which also does not provide a full representation of all UN Member states, and which is also limited with respect to low and least-developed countries. Third, some reporting biases in the existing data set are possible, since variation within countries and between respondents are not addressed. Notwithstanding these potential limitations, we find the two results are particularly interesting when considering actual debates on measuring SDG indicator 6.3.2 on good ambient water quality. This indicator is measured using the core parameters DO, EC, pH, as well as a measure of nitrogen and phosphorus (UNEP 2018). UN Water (2018b, p. 63) highlights that the ‘selected core parameters for indicator 6.3.2 are simple to measure and are a good starting point for countries with less-developed monitoring capacities’. While it may be true that the selected core parameters are relatively easy to measure (in contrast to biological indicators), the results of the correlation analysis suggest that less-developed countries are slightly challenged by the measurement of the chemical water quality parameters nitrogen and phosphorus. This is also in line with recent results of the last monitoring cycle of SDG 6.3.2, revealing significant data gaps and limited numbers of monitoring stations in least-developed countries due to financial reasons. In terms of nitrogen and phosphorus, this may partly go back to the fact that they have not—in contrast to DO, EC and pH—been part of water quality monitoring standards for so long. This again may reflect that gross organic pollution is still often the major water quality issue in many low HDI countries. Nutrients—as being more ecosystem than human health related—are then more a second stage of pollution that becomes problematic only once organic pollution has been addressed.

Achieving a global estimate of water pollution requires strategies to address human development-induced challenges for water quality monitoring. A first general recommendation is to monitor quality parameters that are particularly easy to measure. The difference in measurement challenges between the five key parameters suggest that slight differences in the complexity of measurement methodologies can already have significant effects on the ability of responsible actors to apply these parameters. States may consider, for instance, monitoring easier to measure parameters such as total oxidized N or orthophosphate in case it makes sense in their respective case. These latter two parameters do still normally require laboratory facilities and skilled analysts to achieve accurate measures, making them more demanding than DO, pH and EC which can be measured accurately in the field by unskilled fieldworkers using relatively cheap sensors. More accessible, field-based, citizen-led approaches to monitoring nitrate and orthophosphate using smartphones are becoming available (e.g. FreshWaterWatch 2020; AKVO 2020; Hadj-Hammou et al. 2017; Quinlivan et al. 2020). Moreover, it is up for debate if priority should be set on (i) easy to measure parameters that may then also easily be comparable on a global scale or (ii) on supporting states in monitoring those parameters that are already part of existing monitoring programmes of states.

Thus, a second promising way forward is to increase ‘education for action’ (Irvine et al. 2015), especially based on targeted capacity development (CD) activities for less and least-developed countries. Such CD measures refer to (i) the overarching institutional capacity for water quality monitoring; (ii) the choice of water quality parameters; and, in particular (iii) the actual measurement of the water quality, starting from monitoring, to analytics and then data handling and analysis of, in this case, the core parameters for monitoring SDG indicator 6.3.2. Such measures may be organized globally within the Global Environment Monitoring System for Freshwater (GEMS/Water) (UNEP 2014, Resolution 1.9) or bilaterally in the frame of twinning between more and less-developed states, such as, respectively, EU and third countries (e.g. Harmsen 2015). Such activities should be in line with, and complement, existing tools or guidelines to support water quality monitoring by public authorities (e.g. Behmel et al. 2016; UN Water 2015; UNU-EHS and UNEP 2016). Capacity development activities may also be directed to different addressees such as public authorities on a national or river basin level (Hagemann et al. 2014).

In addition to a thought-out selection process and targeted capacity development measures, addressing the financial dimension is critical. In line with our cost-benefit rationale, international organisations and environmental non-governmental organisations may consistently raise awareness on the role of water quality monitoring for human development. Such awareness raising campaigns may help to increase the perceived benefits of monitoring and thus also to turn the cost-benefit ratio in favour of water quality monitoring, ultimately resulting in higher investments of less-developed countries in water quality monitoring. Besides, the international community may reduce actual costs of water quality monitoring by creating funds for supporting water quality monitoring for states which are willing but not able to invest in monitoring networks. This may be accomplished using existing bi-lateral or internal development support mechanisms.

Finally, states may envision alternative methodologies for water quality monitoring, going beyond the more state-centric traditional approach in monitoring. Much discussed options here are Earth observation as well as the involvement of citizens and the private sector in water quality monitoring (e.g. UN Water 2018a, b; Carvalho et al. 2019). However, such approaches all have their drawbacks, limiting their role in addressing the identified challenges in water quality monitoring. Remote sensing, for instance, is not appropriate for all relevant water quality parameters, requires highly skilled personnel that can hinder application in less-developed countries and, at some point, requires validation through ‘ground truthing’ (Dörnhöfer and Oppelt 2016; Gholizadeh et al. 2016). Involving citizens in water quality monitoring may, however, be a particularly fruitful way forward, given the motivation of citizens for good drinking water quality and other environmental or ecosystem services. Such projects will have to be well-designed to guarantee reliable data in the long run, including requirements for formal institutions, retaining citizens in monitoring programmes and related processes and mechanisms such as funding, feedback culture and so forth (Graham et al. 2004; Conrad and Hilchey 2011; Jollymore et al. 2017; Kim et al. 2018).

Future research on the role of human development in water quality monitoring may address which type or combination of strategies may be best suited to overcome the negative implications of the HDI. Such analyses may include a larger and/or different set of countries, to further validate results of the statistical analyses, which may also be biased due to a high number of responses with regard to countries with a (very) high human development. Such analysis may also ensure the quality of answers of respondents, e.g. by considering answers from responsible public authorities only. Future research may also deepen our analyses, by further including questions of data management, assessment and interpretation of data, or by considering different and emerging methodologies for monitoring (e.g. Angelescu et al. 2019; Rérolle et al. 2019). Also, the inclusion of qualitative data on the challenges of water quality monitoring may help to better understand the mechanisms beyond such negative relations between HDI and the application of water quality parameters. Finally, future research should analyse how specific dimensions of human development correlate with specific challenges such as a lack of human or financial resources. Such research is needed to support enhanced global water quality monitoring.

## Electronic supplementary material

ESM 1 (XLSX 51 kb)

ESM 2 (DOCX 17 kb)

ESM 3 (DOCX 58 kb)

ESM 4 (XLSX 155 kb)

## Footnotes

1 Water quality indicators refer here to single parameters to measure water quality, such as phosphorus, turbidity or pH. Indicators are thus different from indices that refer to combinations of indicators based on set formulas (Lumb et al. 2011; Tirkey et al. 2015).

2 Monitoring refers to the actual data gathering process, whereas analytics refers to laboratory activities, and the subsequent analysis to calculations based on the analytical results.

## References

[CR1] Abbasi T, Abbasi SA (2012). Water quality indices.

[CR2] AbuZeid, K., Elrawady, M., CEDARE, Arab Water Council (2014). “2nd Arab state of the water report – 2012”, Water Resources Management Program – CEDARE & Arab Water Council. http://www.arabwatercouncil.org/images/Publications/Arab_state/2nd_Arab_State_of_the_Water_Report.pdf. Accessed 25.06.2019.

[CR3] AKVO. Online: https://akvo.org/. Accessed 19.02.2020.

[CR4] Angelescu DE, Huynh V, Hausot A, Yalkin G, Plet V, Mouchel J-M, Guérin-Rechdaoui S, Azimi S, Rocher V (2019). Autonomous system for a rapid field quantification of Escherichia coli in surface waters. Journal of Applied Microbiology.

[CR5] Beck L, Bernauer T, Kalbhenn A (2010). Environmental, political, and economic determinants of water quality monitoring in Europe. Water Resources Research.

[CR6] Behmel S, Damour M, Ludwig R, Rodriguez MJ (2016). Water quality monitoring strategies – a review and future perspectives. Science of the Total Environment.

[CR7] Bharti N, Katyal D (2011). Water quality indices used for surface water vulnerability assessment. International Journal of Environmental Sciences.

[CR8] Carvalho L, Mackay EB, Cardoso AC, Baattrup-Pedersen A, Birk S, Blackstock KL, Borics G, Borja A, Feld CK, Ferreira MT, Globevnik L (2019). Protecting and restoring Europe’s waters: an analysis of the future development needs of the Water Framework Directive. Science of the Total Environment.

[CR9] Conrad CC, Hilchey KG (2011). A review of citizen science and community-based environmental monitoring: issues and opportunities. Environmental Monitoring and Assessment.

[CR10] Council of the European Communities (2000). Directive 2000/60/EC of the European Parliament and of the Council of 23 October 2000 establishing a framework for community action in the field of water policy. Official Journal of the European Communities.

[CR11] Delaire C, Peletz R, Kumpel E, Kisiangani J, Bain R, Khush R (2017). How much will it cost to monitor microbial drinking water quality in sub-Saharan Africa?. Journal of Environmental Science & Technology.

[CR12] Dörnhöfer K, Oppelt N (2016). Remote sensing for lake research and monitoring – recent advances. Ecological Indicators.

[CR13] Dunlap R, York R (2008). The globalization of environmental concern and the limits of the postmaterialist values explanation: evidence from four multinational surveys. The Sociological Quarterly.

[CR14] European Environment Agency (EEA) (2012): European waters – assessment of status and pressures. EEA Report No 8/2012.

[CR15] Evans AEV, Hanjra MA, Jiang Y, Qadir M, Drechsel P (2012). Water quality: assessment of the current situation in Asia. International Journal of Water Resources Development.

[CR16] Federal Water pollution control act [As Amended Through P.L. 107–303, November 27, 2002]. https://www.epa.gov/sites/production/files/2017-08/documents/federal-water-pollution-control-act-508full.pdf. Referenced via Water Quality Standards Academy Water Quality Standards Academy California (2008). Beneficial Uses. California Perspective. Online: https://www.waterboards.ca.gov/academy/courses/wqstandards/materials/mod3/cabenuses.pdf.

[CR17] Ferrero Giuliana, Setty Karen, Rickert Bettina, George Shannan, Rinehold Angella, DeFrance Jennifer, Bartram Jamie (2019). Capacity building and training approaches for water safety plans: A comprehensive literature review. International Journal of Hygiene and Environmental Health.

[CR18] FreshWaterWatch. Online: https://freshwaterwatch.thewaterhub.org/. Accessed 19.02.2020.

[CR19] Gholizadeh MH, Melesse AM, Reddi L (2016). A comprehensive review on water quality parameters estimation using remote sensing techniques. Sensors.

[CR20] Graham PM, Dickens CWS, Taylor RJ (2004). miniSASS—A novel technique for community participation in river health monitoring and management. African Journal of Aquatic Science.

[CR21] Hadj-Hammou J, Loiselle S, Ophof D, Thornhill I (2017). Getting the full picture: assessing the complementarity of citizen science and agency monitoring data. PLoS One.

[CR22] Hagemann N, Klauer B, Moynihan RM, Leidel M, Scheifhacken N (2014). The role of institutional and legal constraints on river water quality monitoring in Ukraine. Environmental Earth Sciences.

[CR23] Harmsen, J. (2015). National monitoring implementation plan: EU twinning project capacity building on water quality monitoring. *European Union*.

[CR24] Horowitz AJ (2013). A review of selected inorganic surface water quality-monitoring practices: are we really measuring what we think, and if so, are we doing it right?. Environmental Science & Technology.

[CR25] Ibisch RB, Leidel M, Niemann S, Hornidge A-K, Goedert R, Borchardt D, Bogardi J, Ibisch R (2016). Capacity development for integrated water resources management: lessons learned from applied research projects. Integrated water resources management: concept, research and implementation.

[CR26] Irvine K, Weigelhofer G, Popescu I, Pfeiffer E, Păun A, Drobot R, Gettel G, Staska B, Stanica A, Hein T, Habersack H (2015). Educating for action: aligning skills with policies for sustainable development in the Danube river basin. Science of the Total Environment.

[CR27] Irvine K, Castello L, Junqueira A, Moulton T (2016). Linking ecology with social development for tropical aquatic conservation. Aquatic Conservation: Marine and Freshwater Ecosystem.

[CR28] Jollymore A, Hainesa MJ, Satterfield T, Johnson MS (2017). Citizen science for water quality monitoring: data implications of citizen perspectives. Journal of Environmental Management.

[CR29] Kim, J., Kirschke, S., Avellán, T. (2018). Well-designed citizen science projects can help monitor SDG 6. *SDG Knowledge Hub*, n/a–n/a.

[CR30] Leidel M, Niemann S, Hagemann N (2012). Capacity development as a key factor for integrated water resources management (IWRM): improving water management in the Western Bug River Basin, Ukraine. Environmental Earth Sciences.

[CR31] Lumb A, Sharma TC, Bibeault J-F (2011). A review of genesis and evolution of Water Quality Index (WQI) and some future directions. Water Quality Exposure and Health.

[CR32] Marquart-Pyatt ST (2012). Contextual influences on environmental concerns cross-nationally: a multilevel investigation. Social Science Research.

[CR33] Neuenfeldt, S., Kirschke, D., & Franke, C. (2012). *Was sagt der Human Development Index über Entwicklung aus*? Kritik und Erweiterung auf der Grundlage eines faktoranalytischen Ansatzes. Working paper 91, HU Berlin.

[CR34] Peletz R, Kisiangani J, Bonham M, Ronoh P, Delaire C, Kumpel E, Marks S, Khush R (2018). Why do water quality monitoring programs succeed or fail? A qualitative comparative analysis of regulated testing systems in sub-Saharan Africa. International Journal of Hygiene and Environmental Health.

[CR35] Poikane S, Birk S, Böhmer J, Carvalho L, de Hoyos C, Gassner H, Hellsten S, Kelly M, Solheim AL, Olin M, Pall K (2015). A hitchhiker’s guide to European lake ecological assessment and intercalibration. Ecological Indicators.

[CR36] Quinlivan L, Chapman DV, Sullivan T (2020). Validating citizen science monitoring of ambient water quality for the United Nations sustainable development goals. Science of the Total Environment.

[CR37] Rahman Z, Crocker J, Chang K, Khush R, Bartram J (2011). A comparative assessment of institutional frameworks for managing drinking water quality. Journal of Water, Sanitation and Hygiene for Development.

[CR38] Rérolle, V., Angelescu, D., Hausot, A., Ea, P., Lefèvre, N., Provost, C., & Labaste, M. (2019). Development of a novel hybrid pH sensor for deployment on autonomous profiling platforms. In *OCEANS 2019-Marseille* (pp. 1–8). IEEE.

[CR39] Samimi AJ, Kashefi A, Salatin P, Lashkarizadeh M (2011). Environmental performance and HDI: evidence from countries around the world. Middle-East Journal of Scientific Research.

[CR40] Srebotnjaka T, Carr G, de Sherbinin A, Rickwoodd C (2012). A global Water Quality Index and hot-deck imputation of missing data. Ecological Indicators.

[CR41] Steynberg MC (2002). Drinking water quality assessment practices: an international perspective. Water Science and Technology: Water Supply.

[CR42] Tirkey P, Bhattacharya T, Chakraborty S (2015). Water quality indices – important tools for water quality assessment: a review. International Journal of Advances in Chemistry.

[CR43] UN Water (2015). *Compendium of water quality regulatory frameworks: which water for which use?* www.iwa-network.org/which-water-for-which-use. Accessed 25.06.2019.

[CR44] UN Water (2018a). *Step-by-step monitoring methodology for indicator 6.3.2 ‘Proportion of bodies of water with good ambient water quality’*. Online: http://www.unwater.org/publications/step-step-methodology-monitoring-water-quality-6-3-2/.

[CR45] UN Water (2018b). *Sustainable development goal 6*. Synthesis report on water and sanitation. Online: http://www.unwater.org/publication_categories/sdg-6-synthesis-report-2018-on-water-and-sanitation/. Accessed 15 Aug 2018.

[CR46] UN Water (2019). *National systems to support drinking-water, sanitation and hygiene: global status report 2019*. Online: https://apps.who.int/iris/bitstream/handle/10665/326444/9789241516297-eng.pdf?ua=1. Assessed 20 Jan 2019.

[CR47] UNDP (2016). Human development report 2016. Human Development for Everyone.

[CR48] UNEP (2010). Africa water atlas. Division of early warning and assessment (DEWA).

[CR49] UNEP (2012). Status report on the application of integrated approaches to water resources management.

[CR50] UNEP (2014). *Resolutions and decisions adopted by the United Nations environment assembly of the United Nations environment Programme at its first session on 27 June 2014*. http://wedocs.unep.org/bitstream/handle/20.500.11822/17285/K1402364.pdf?sequence=3&isAllowed=y. Accessed 25.06.2019.

[CR51] UNEP (2016). A snapshot of the world’s water quality: towards a global assessment.

[CR52] UNEP (2018). Progress on ambient water quality. *Piloting the monitoring methodology and initial findings for SDG 6 indicator 6.3.2*. http://www.unwater.org/publications/progress-on-ambient-water-quality-632/. Accessed 25.06.2019.

[CR53] UNICEF/WHO (2019). Progress on household drinking water, sanitation and hygiene 2000–2017. Special focus on inequalities.

[CR54] UNU-EHS and UNEP (2016). International water quality guidelines for ecosystems (IWQGES). *Draft for regional consultations*. http://web.unep.org/sites/default/files/Documents/20160315_iwqges_pd_final.pdf. Accessed 15 March 2016.

[CR55] UNU-FLORES (2017). *Call for water professionals to fill out survey on water quality indicators*. https://flores.unu.edu/en/about/media/releases/call-for-water-professionals-to-fill-out-survey-on-water-quality-indicators.html#info. Accessed 25.06.2019.

[CR56] When de Montalvo U, Alaerts G (2013). Leadership in knowledge and capacity development in the water sector: A status review. Water Policy.

[CR57] WHO (World Health Organization) (2011). Guidelines for drinking-water Quality.

[CR58] WHO/UNICEF (2017). Progress on drinking water, sanitation and hygiene: 2017 update and SDG baselines.

[CR59] WWAP (World Water Assessment Programme) (2012). The United Nations world water development report 4: managing water under uncertainty and risk.

