# Supplementary material for: Capacity challenges in water quality monitoring: understanding the role of human development
Source: Environ Monit Assess. 2020 Apr 19;192(5):298. doi: 10.1007/s10661-020-8224-3 (PMC7167377; doi:10.1007/s10661-020-8224-3)
Supplement: Supplementary file 2 — (DOCX 17 kb) [file 10661_2020_8224_MOESM2_ESM.docx]

**Networks for spreading the questionnaire on water quality indicators**

| **No** | **Network** | **Target group** | |
| --- | --- | --- | --- |
|  |  | **Type of actors** | **Regional scope** |
| 1 | Arab Countries Water Utilities Association (ACWUA) | Practitioners (water utilities) | Arab countries |
| 2 | Australian Water Association | Scientists and practitioners (various types) | Australia |
| 3 | European Topic Centre | Practitioners (public authorities) | European countries |
| 4 | Contact points of the funding initiative IWRM of the German Ministry of Education and Research | Scientists and practitioners | International |
| 5 | Water network of the *Deutsche Gesellschaft für internationale Zusammenarbeit und Entwicklung* (GIZ) | Practitioners (e.g. development cooperation, government officials) | International (focus developing countries) |
| 6 | Global Water Partnership (GWP) | Practitioners (e.g. water utilities) | International |
| 7 | Global Waters Operators‘ Partnerships Alliance (GWOPA) | Practitioners (water utilities) | International |
| 8 | International Water Association | Scientists (mainly natural science) | International |
| 9 | UNU-FLORES network | Scientists and practitioners (various types) | International |
| 10 | UN Water | Practitioners (list 8)) | International |
| 11 | Water Science Alliance | Scientists (mainly natural scientists) | Germany |
