# Supplementary material for: Capacity challenges in water quality monitoring: understanding the role of human development
Source: Environ Monit Assess. 2020 Apr 19;192(5):298. doi: 10.1007/s10661-020-8224-3 (PMC7167377; doi:10.1007/s10661-020-8224-3)
Supplement: Supplementary file 3 — (DOCX 58 kb) [file 10661_2020_8224_MOESM3_ESM.docx]

**Questionnaire on Water Quality Indicators and Indices**

*Dear Sir or Madam,*

*This questionnaire aims to analyse the application of water quality indicators and indices for measuring water quality in practice. It is a joint effort by scientists and practitioners in order to better understand challenges for achieving water-related sustainability goals. Results will help experts in designing relevant capacity development programs for measuring water quality in the future.*

*By water quality, we mean both human health and ecosystem related aspects.* ***Indicators*** *refer here to single parameters to measure water quality (e.g. phosphorus, pH) whereas* ***indices*** *refer to combinations of indicators (e.g. WATQI which includes dissolved oxygen, electrical conductivity, pH, total nitrogen and total phosphorus).*

*Please answer the questions based on your best knowledge in a certain case. Cases can refer here to different types of waters (e.g. groundwater or flowing water) at various scales (e.g. water body, basin, or nation state). Answering the questionnaire will require about 30 minutes. Please contact Sabrina Kirschke from UNU-FLORES (kirschke@unu.edu) if there are any questions regarding the questionnaire.*

*Thank you for your support!*

*UNU-FLORES*

**1. General information**

**1.1 Please indicate if you provide an individual or a collective answer.**

My personal answer

Answer for a group (e.g. the answer for my organization)

**1.2 From the stakeholder category below, please select the one that represents you best.**

Government official

Civil society

Private sector

Academic

Development agency

Other. *Please specify*

**1.3 Please indicate your experience related to water quality indicators and indices.**

Up to 3 years

4 to 6 years

More than 7 years

- 1. **Please indicate your gender.**

female

male

- 1. **Please indicate your nationality.**

-- Select --

- 1. **Please indicate the country your answers refer to.**

-- Select --

- 1. **Please indicate the scale your answers refer to.**

Water body

(Sub-)basin.

Region.

Other. *Please specify*

- 1. **Please indicate the type of water your case refers to.**

Rivers

Lakes

Estuaries

Groundwater

**2. General use of indices and indicators in your case**

**2.1 What are the main driving factors for addressing water quality challenges in your case?**

**Propagation of fish, shellfish & wildlife** not important factor 1  2  3  4  very important factor;  Don’t know

(Refers to fishing purposes and biodiversity)

**Recreation in & on the water** not important factor 1  2  3  4  very important factor;  Don’t know

(Refers to tourism)

**Public water supplies**  not important factor 1  2  3  4  very important factor;  Don’t know

(Refers to providing drinking water)

**Agricultural use**  not important factor 1  2  3  4  very important factor;  Don’t know

(Refers to the cultivation of crops for food and energy supply)

**Industrial use**  not important factor 1  2  3  4  very important factor;  Don’t know

(Refers to the production of various goods)

**Navigation**  not important factor 1  2  3  4  very important factor;  Don’t know

(Refers to the transport of goods and people)

Other (*Please specify)* not important factor 1  2  3  4  very important factor;  Don’t know

**2.2 How difficult is it to identify the most relevant water quality *index* for your case?**

(Indices refer here to combinations of indicators (e.g. WATQI which includes dissolved oxygen, electrical conductivity, pH, total nitrogen and total phosphorus))

not difficult 1  2  3  4  very difficult;  Don’t know

**2.3 How difficult is it to identify the most relevant water quality *indicators* for your specific context?**

(Indicators refer here to single parameters to measure water quality (e.g. phosphorus, pH))

not difficult 1  2  3  4  very difficult;  Don’t know

**2.4 Which water quality index is used in your case?**

British Columbia Water quality Index

Canadian Council of Ministers of the Environment Water Quality Index

National Sanitation Foundation Water Quality Index

Global Water Quality Index (GEMS WQI)

Water quality indices are not used in my case.

I don’t know.

Other (*Please specify)*

**2.5 How important are the following indicator groups in your case in order to measure water quality?**

**Physical indicators**  not important 1  2  3  4  very important;  Don’t know

(e.g. temperature, light penetration (Secchi depth))

**Chemical indicators**  not important 1  2  3  4  very important;  Don’t know

(e.g. nutrients such as nitrogen and phosphorus, *excluding* contaminants (of emerging concern))

**Contaminants (of emerging concern)** not important 1  2  3  4  very important;  Don’t know

(e.g. toxicants such as heavy metals, pesticides, PCBs and VOCs)

**Biological Indicators** not important 1  2  3  4  very important;  Don’t know

(e.g. fish, invertebrates, algae, macrophytes, *excluding bacteria*)

**Intestinal health Indicators** not important 1  2  3  4  very important;  Don’t know

(e.g. microbial pollutants such as E. coli, cholera, Gardia species)

**2.6 Which of the following five indicators are used in your case in order to measure water quality?**

**Dissolved oxygen (DO)** Used  Not used  Don’t know

**Electrical conductivity (EC)** Used  Not used  Don’t know

**pH** Used  Not used  Don’t know

**Total phosphorus (P)** Used  Not used  Don’t know

**Total nitrogen (N)** Used  Not used  Don’t know

**Other (*Please specify)***  Used  Not used  Don’t know

**3.1 Measuring dissolved oxygen (DO)**

***Please fill in if the indicator is used in your case.***

**3.1.1 Which standard is used to measure dissolved oxygen (DO) in your case?**

American Public Health Organization (APHA) standard

United States Environmental Protection Agency (EPA) standard

International Organization for Standardization (ISO) standard

Do not use published standards.

Don’t know.

Other *(Please specify)*

**3.1.2 How challenging is the application of dissolved oxygen (DO) in your case?**

| **Procedures** | **Current practices** |
| --- | --- |
| **Monitoring challenges** |  |
| **Providing the technical equipment for monitoring**  (e.g. buckets, bottles, test kits, etc.) | not challenging 1  2  3  4  very challenging;  Don’t know |
| **Providing human skills for monitoring**  (e.g. recruiting people educated in monitoring techniques) | not challenging 1  2  3  4  very challenging;  Don’t know |
| **Providing financial means for monitoring**  (e.g. for the equipment, human resources etc.) | not challenging 1  2  3  4  very challenging;  Don’t know |
| **Analytical challenges** |  |
| **Providing technical equipment for analytics**  (e.g. chemical reagents, sensors, manometers, etc.) | not challenging 1  2  3  4  very challenging;  Don’t know |
| **Providing human skills for analytics**  (e.g. recruiting people educated in analytical techniques) | not challenging 1  2  3  4  very challenging;  Don’t know |
| **Providing financial means for analytics**  (e.g. for instruments, human resources etc.) | not challenging 1  2  3  4  very challenging;  Don’t know |
| **Challenges related to data handling / analysis** |  |
| **Providing technical equipment for data handling/ analysis**  (e.g. software for statistical analysis) | not challenging 1  2  3  4  very challenging;  Don’t know |
| **Providing human skills for data handling/ analysis**  (e.g. recruiting people educated in statistics) | not challenging 1  2  3  4  very challenging;  Don’t know |
| **Providing financial means for data handling/ analysis**  (e.g. for software, human resources etc.) | not challenging 1  2  3  4  very challenging;  Don’t know |
| **Transferring data to relevant groups**  (e.g. making data accessible or usable for practitioners) | not challenging 1  2  3  4  very challenging;  Don’t know |

**3.2 Measuring electrical conductivity (EC)**

***Please fill in if the indicator is used in your case.***

**3.2.1 Which standard is used to measure electrical conductivity (EC) in your case?**

American Public Health Organization (APHA) standard

United States Environmental Protection Agency (EPA) standard

International Organization for Standardization (ISO) standard

Do not use published standards.

Don’t know.

Other (*Please specify)*

**3.2.2 How challenging is the application of electrical conductivity (EC) in your case?**

| **Procedures** | **Current practices** |
| --- | --- |
| **Monitoring challenges** |  |
| **Providing the technical equipment for monitoring**  (e.g. buckets, bottles, test kits, etc.) | not challenging 1  2  3  4  very challenging;  Don’t know |
| **Providing human skills for monitoring**  (e.g. recruiting people educated in monitoring techniques) | not challenging 1  2  3  4  very challenging;  Don’t know |
| **Providing financial means for monitoring**  (e.g. for the equipment, human resources etc.) | not challenging 1  2  3  4  very challenging;  Don’t know |
| **Analytical challenges** |  |
| **Providing technical equipment for analytics**  (e.g. chemical reagents, sensors, manometers, etc.) | not challenging 1  2  3  4  very challenging;  Don’t know |
| **Providing human skills for analytics**  (e.g. recruiting people educated in analytical techniques) | not challenging 1  2  3  4  very challenging;  Don’t know |
| **Providing financial means for analytics**  (e.g. for instruments, human resources etc.) | not challenging 1  2  3  4  very challenging;  Don’t know |
| **Challenges related to data handling / analysis** |  |
| **Providing technical equipment for data handling/ analysis**  (e.g. software for statistical analysis) | not challenging 1  2  3  4  very challenging;  Don’t know |
| **Providing human skills for data handling/ analysis**  (e.g. recruiting people educated in statistics) | not challenging 1  2  3  4  very challenging;  Don’t know |
| **Providing financial means for data handling/ analysis**  (e.g. for software, human resources etc.) | not challenging 1  2  3  4  very challenging;  Don’t know |
| **Transferring data to relevant groups**  (e.g. making data accessible or usable for practitioners) | not challenging 1  2  3  4  very challenging;  Don’t know |

**3.3 Measuring pH**

***Please fill in if the indicator is used in your case.***

**3.3.1 Which standard is used to measure pH in your case?**

American Public Health Organization (APHA) standard

United States Environmental Protection Agency (EPA) standard

International Organization for Standardization (ISO) standard

Do not use published standards.

Don’t know.

Other *(Please specify)*

**3.3.3 How challenging is the application of pH in your case?**

| **Procedures** | **Current practices** |
| --- | --- |
| **Monitoring challenges** |  |
| **Providing the technical equipment for monitoring**  (e.g. buckets, bottles, test kits, etc.) | not challenging 1  2  3  4  very challenging;  Don’t know |
| **Providing human skills for monitoring**  (e.g. recruiting people educated in monitoring techniques) | not challenging 1  2  3  4  very challenging;  Don’t know |
| **Providing financial means for monitoring**  (e.g. for the equipment, human resources etc.) | not challenging 1  2  3  4  very challenging;  Don’t know |
| **Analytical challenges** |  |
| **Providing technical equipment for analytics**  (e.g. chemical reagents, sensors, manometers, etc.) | not challenging 1  2  3  4  very challenging;  Don’t know |
| **Providing human skills for analytics**  (e.g. recruiting people educated in analytical techniques) | not challenging 1  2  3  4  very challenging;  Don’t know |
| **Providing financial means for analytics**  (e.g. for instruments, human resources etc.) | not challenging 1  2  3  4  very challenging;  Don’t know |
| **Challenges related to data handling / analysis** |  |
| **Providing technical equipment for data handling/ analysis** (e.g. software for statistical analysis) | not challenging 1  2  3  4  very challenging;  Don’t know |
| **Providing human skills for data handling/ analysis**  (e.g. recruiting people educated in statistics) | not challenging 1  2  3  4  very challenging;  Don’t know |
| **Providing financial means for data handling/ analysis**  (e.g. for software, human resources etc.) | not challenging 1  2  3  4  very challenging;  Don’t know |
| **Transferring data to relevant groups**  (e.g. making data accessible or usable for practitioners) | not challenging 1  2  3  4  very challenging;  Don’t know |

**3.4 Measuring Total phosphorus (P)**

***Please fill in if the indicator is used in your case.***

**3.4.1 Which standard is used to measure Total Phosphorus (P) in your case?**

American Public Health Organization (APHA) standard

United States Environmental Protection Agency (EPA) standard

International Organization for Standardization (ISO) standard

Do not use published standards.

Don’t know.

Other *(Please specify)*

**3.4.2 How challenging is the application of Total phosphorus (P) in your case?**

| **Procedures** | **Current practices** |
| --- | --- |
| **Monitoring challenges** |  |
| **Providing the technical equipment for monitoring**  (e.g. buckets, bottles, test kits, etc.) | not challenging 1  2  3  4  very challenging;  Don’t know |
| **Providing human skills for monitoring**  (e.g. recruiting people educated in monitoring techniques) | not challenging 1  2  3  4  very challenging;  Don’t know |
| **Providing financial means for monitoring**  (e.g. for the equipment, human resources etc.) | not challenging 1  2  3  4  very challenging;  Don’t know |
| **Analytical challenges** |  |
| **Providing technical equipment for analytics**  (e.g. chemical reagents, sensors, manometers, etc.) | not challenging 1  2  3  4  very challenging;  Don’t know |
| **Providing human skills for analytics**  (e.g. recruiting people educated in analytical techniques) | not challenging 1  2  3  4  very challenging;  Don’t know |
| **Providing financial means for analytics**  (e.g. for instruments, human resources etc.) | not challenging 1  2  3  4  very challenging;  Don’t know |
| **Challenges related to data handling / analysis** |  |
| **Providing technical equipment for data handling/ analysis**  (e.g. software for statistical analysis) | not challenging 1  2  3  4  very challenging;  Don’t know |
| **Providing human skills for data handling/ analysis**  (e.g. recruiting people educated in statistics) | not challenging 1  2  3  4  very challenging;  Don’t know |
| **Providing financial means for data handling/ analysis**  (e.g. for software, human resources etc.) | not challenging 1  2  3  4  very challenging;  Don’t know |
| **Transferring data to relevant groups**  (e.g. making data accessible or usable for practitioners) | not challenging 1  2  3  4  very challenging;  Don’t know |

**3.5 Measuring Total nitrogen (N)**

***Please fill in if the indicator is used in your case.***

**3.5.1 Which standard is used to measure total nitrogen (N) in your case?**

American Public Health Organization (APHA) standard

United States Environmental Protection Agency (EPA) standard

International Organization for Standardization (ISO) standard

Do not use published standards.

Don’t know.

Other *(Please specify)*

**3.5.2 How challenging is the application of total nitrogen (N) in your case?**

| **Procedures** | **Current practices** |
| --- | --- |
| **Monitoring challenges** |  |
| **Providing the technical equipment for monitoring**  (e.g. buckets, bottles, test kits, etc.) | not challenging 1  2  3  4  very challenging;  Don’t know |
| **Providing human skills for monitoring**  (e.g. recruiting people educated in monitoring techniques) | not challenging 1  2  3  4  very challenging;  Don’t know |
| **Providing financial means for monitoring**  (e.g. for the equipment, human resources etc.) | not challenging 1  2  3  4  very challenging;  Don’t know |
| **Analytical challenges** |  |
| **Providing technical equipment for analytics**  (e.g. chemical reagents, sensors, manometers, etc.) | not challenging 1  2  3  4  very challenging;  Don’t know |
| **Providing human skills for analytics**  (e.g. recruiting people educated in analytical techniques) | not challenging 1  2  3  4  very challenging;  Don’t know |
| **Providing financial means for analytics**  (e.g. for instruments, human resources etc.) | not challenging 1  2  3  4  very challenging;  Don’t know |
| **Challenges related to data handling / analysis** |  |
| **Providing technical equipment for data handling/ analysis**  (e.g. software for statistical analysis) | not challenging 1  2  3  4  very challenging;  Don’t know |
| **Providing human skills for data handling/ analysis**  (e.g. recruiting people educated in statistics) | not challenging 1  2  3  4  very challenging;  Don’t know |
| **Providing financial means for data handling/ analysis**  (e.g. for software, human resources etc.) | not challenging 1  2  3  4  very challenging;  Don’t know |
| Transferring data to relevant groups  (e.g. making data accessible or usable for practitioners) | not challenging 1  2  3  4  very challenging;  Don’t know |

**3.6 Measuring other indicators**

| ***Please specify a particularly important water quality indicator in your case. Please relate your following answers to this indicator.*** |
| --- |
|  |

**3.6.1 Which standard is used to measure other indicators in your case?**

American Public Health Organization (APHA) standard

United States Environmental Protection Agency (EPA) standard

International Organization for Standardization (ISO) standard

Do not use published standards.

Don’t know.

Other *(Please specify)*

**3.6.2 How challenging is the application of other indicators in your case?**

| **Procedures** | **Current practices** |
| --- | --- |
| **Monitoring challenges** |  |
| **Providing the technical equipment for monitoring**  (e.g. buckets, bottles, test kits, etc.) | not challenging 1  2  3  4  very challenging;  Don’t know |
| **Providing human skills for monitoring**  (e.g. recruiting people educated in monitoring techniques) | not challenging 1  2  3  4  very challenging;  Don’t know |
| **Providing financial means for monitoring**  (e.g. for the equipment, human resources etc.) | not challenging 1  2  3  4  very challenging;  Don’t know |
| **Analytical challenges** |  |
| **Providing technical equipment for analytics**  (e.g. chemical reagents, sensors, manometers, etc.) | not challenging 1  2  3  4  very challenging;  Don’t know |
| **Providing human skills for analytics**  (e.g. recruiting people educated in analytical techniques) | not challenging 1  2  3  4  very challenging;  Don’t know |
| **Providing financial means for analytics**  (e.g. for instruments, human resources etc.) | not challenging 1  2  3  4  very challenging;  Don’t know |
| **Challenges related to data handling / analysis** |  |
| **Providing technical equipment for data handling/ analysis**  (e.g. software for statistical analysis) | not challenging 1  2  3  4  very challenging;  Don’t know |
| **Providing human skills for data handling/ analysis**  (e.g. recruiting people educated in statistics) | not challenging 1  2  3  4  very challenging;  Don’t know |
| **Providing financial means for data handling/ analysis**  (e.g. for software, human resources etc.) | not challenging 1  2  3  4  very challenging;  Don’t know |
| **Transferring data to relevant groups**  (e.g. making data accessible or usable for practitioners) | not challenging 1  2  3  4  very challenging;  Don’t know |

**4. Governance and management challenges in measuring water quality**

**4.1 Is water quality information readily accessible in your case?**

Yes  No  Don’t know

**4.2 Are there obligatory rules to measure water quality in your case?**

Yes  No  Don’t know

**4.3 Are there monitoring strategies to measure water quality in your case?**

Yes  No  Don’t know

**4.4 Are there responsible authorities to measure water quality in your case?**

Yes  No  Don’t know

**4.5 Are there enough institutional capacities to measure water quality in your case?**

Yes  No  Don’t know

**4.6 What are the main management challenges related to the application of water quality indices and indicators in your case?**

*Please indicate the three most important management challenges related to the proper application of water quality indices and indicators in your case. The challenges can refer to the categories above or to other types of challenges.*

**5. Additional comments**

**5.1 Please add additional comments related to the topic of water quality indicators and indices. Please also specify if these are general comments or if they refer to your case.**

**THANK YOU ☺**
